# ITEPE: A Source Tracing Algorithm for the Microblog

**DOI:** 10.1371/journal.pone.0111380

**Published:** 2014-10-31

**Authors:** Xueyan Zhou, Jing Yang, Zehong Lin, Jianpei Zhang

**Affiliations:** 1 College of Computer Science and Technology, Harbin Engineering University, Harbin, China; 2 College of Engineering, Harbin University, Harbin, China; Beijing University of Posts and Telecommunications, China

## Abstract

Finding the true source of a social network is a crucial component of social network information tracing. Using the new media microblog as an example, this paper provides a source tracing algorithm ITEPE (Initiators and Early Participants Extraction) to solve this problem. First, the cascade (session tree) is built according to the retweeting of a microblog, after which the cascade set (session forest) is clustered by topical relevance. Second, real initiators are identified through the user relationship network and information cascade network. The influence index and conformity index of every node is then iteratively calculated according to text sentiment analysis and information cascades and the early important participants are extracted. Finally, the real initiators and early participants are evaluated through an experiment.

## Introduction

As an online social media tool, the microblog has experienced rapid development in recent times. Microblogs allow users to broadcast messages and share feelings and information at any time, and these messages are automatically pushed to friends' homepages, forming the automatic propagation mechanism. The microblog has become a concentrated expression and reflection of public opinion on the Internet, which largely affects public opinion in general [1]. Although the spread range of messages in daily life is small, the development of social networks has amplified it greatly. For example, public opinion originating on social networks spreads quickly and easily due to diverse information, powerful interactivity and other advantages that traditional media cannot match; however, fuzzy and false information can also appear on social media. Harmful information can be propagated across regions and borders through open-ended communication, particularly reactionary rhetoric attacking social stability. Thus, source tracing research is necessary to quell rumors. The current study focuses on topic detection and tracking. Source tracing research is limited. Information propagated through microblogging spreads quickly, reaching larger audiences and exerting a wider influence than information disseminated through other channels, rendering it difficult to trace. Source tracing and the identification of events and key figures in microblog propagation has become a significant problem in need of a solution. Therefore, quantifying the participation degree of users, confirming the topic source and extracting key persons are the key foci of this paper.

Source tracing includes mining the true initiator and early important participants because some users simply repost or directly copy other's blog messages, with the result that the early participants are sometimes more important than the initiators. To determine these important nodes, this paper presents the ITEPE source tracing algorithm. The forwarded cascades (session tree) are built and the cascade sets (session forest) are then clustered according to topic relevance. Second, the real initiators are determined through a combination of user networks and information cascade networks. The influence index and conformity index of every node are then iteratively calculated according to text sentiment analysis and information cascades and the early important participants are extracted. The real initiators and early participants will be obtained and evaluated through an experiment. Compared to previous studies, the ITEPE algorithm has the ability to trace back to the information source through microblog retweeting cascades. Related research has not been identified.

There have been recent several studies on the social network information diffusion model, and multiple algorithms extract social networks from a group of the most influential nodes. The basic idea is that these nodes will disseminate information more widely, including information dissemination predicted by analyzing the blog information cascade [2], [3]. According to Dabeer [4], who analyzed the factors affecting microblog information dissemination, including information characteristics and the activity, response and out-degree of fan nodes, the information dissemination speed of microblogs is faster than that of traditional blogs and their propagation models differ. Dabeer further proposed a decision making framework based on Markov to measure the effectiveness of information dissemination. Lehmann [5] tracked the hashtag diffusion process in the Twitter network and discovered that epidemic spread models play an important role. Yang [6] predicted the speed, size and scope of microblog information dissemination, and Tsur [7] combined content and network topology by using linear regression to predict the diffusion of information within a given timeframe.

This paper differs from the existing studies in two ways. First, most of the existing algorithms extract and sort the high-influence nodes but do not consider topic relevancy. Yang [8] presented a linear influence model using microblog network relationships to predict the diffusion path and provide the global diffusion capacity of each node. The number of studies focusing on the information source remains relatively small. Recent studies show that the dissemination of information does not depend on the most influential person but on the mutual influence among those likely to be affected [9]. An important conclusion of the study is that early important participants are more influential than the message initiator in the information dissemination cascade. Our research will focus on the traceability research of the microblog information spread cascade, including the true information initiator and early important participants. Second, the topic-based emotion subgraph is proposed to analyze the influence and conformity index in cascade sets to mine the key person.

Retweets from microblogs are the main data mining objects because retweets are the basis of information dissemination, influence analysis, sentiment analysis, topic discovery and evolution, and so on. Therefore, the study of microblog retweeting helps us understand the information diffusion mechanism. Macskassy [10] showed that the majority of users do not necessarily retweet familiar topics. Yang [11] studied the Twitter retweeting mechanism with results indicating that approximately 1/4 of published microblog tweets are retweets of friends' posts. Welch [12] studied the semantic information of the follow and retweeting relationship and found that the latter has stronger topical relevance. Pal and Counts [13] assessed and sorted users' authority using the number of original tweets, participants in the session and retweeting as a primary index; their model used a large calculation quantity Gaussian mixture model to calculate the user influence, which is not suitable for traceability research. Meanwhile, the user influence assessment based on information must address many different languages, dialects, pictures and videos, and so on. Therefore, we must use a simple method to gather accurate information such that only the cascade and the topology are used to study traceability without semantic mining, and only positive and negative emotions are used to assess influence while ignoring the impact of information in different formats.

The influence of microblogs can be understood as a user being affected by other users in such a way as to change his or her behavior. There are two separate approaches to key person extraction in social networks: the approach based on context roles and that based on social network structures. The most common key person extraction methods rely on various centrality measures for each node separately. These algorithms, however, lack a holistic view, and the node position in a social community is determined by its neighborhoods, such as degree prestige and degree centrality. There are also global algorithms, such as proximity prestige, rank prestige, node position, eccentricity and closeness centrality. Many studies have examined various domains (influence spread, public opinion analysis and terrorist group analysis) in this area [14]–[17]. Most existing influence analysis algorithms are improved using traditional algorithms. Cha et al. used indegree, retweets and mentions to measure individual influence on Twitter. Additionally, influence can be measured by diffusion ability, as in Bakshy [19], who used Twitter data URL structures to build the cascade propagation tree and measured node influence on its diffusion range. Steeg [20] suggested a measure of causal relationships between nodes based on the information-theoretic notion of transfer entropy, or information transfer. Most of these methods, however, only consider node influence and ignore the conformity assessment. The purpose of social network traceability is to find the information source. Thus, the information initiators and early important nodes are the main mining objects. However, the existing algorithms cannot meet this demand, so we use the topic-based cascade and users' topologies to confirm the information source by calculating the influence index and conformity index. The result sets include true information initiators and early participants.

## Methods

We introduce some of the related terms and concepts of the microblog information cascade in Table 1.

**Table 1:** Symbol profile.

Symbols	Semantics
*C^i^*	Cascade *i*
*T^i^*	Timestamp when the first message appears in *C^i^*
*φ^i^*	Set of all message that appeared in *C^i^*
*ς*	Topic-based cascade set
*G_T_*	Social network cascade set based on topic T
Φ(*v*)	Node Influence Index
Ω(*v*)	Node Conformity Index
IT_T_	Effective initiator of topic T
EP_T_	Early participants of topic T
KP_T_	Key persons for the sourcing of topic T

### The cascade in microblogs

Users can publish their feelings and ideas via microblogs, which are composed of text, pictures and corresponding comments. A very important element of a post is the comments section, which allows for discussions. The microblog has become an important tool for information dissemination and for this reason, learning more about the microblog information diffusion mechanism is important for the establishment of a new concept of rapid development communication.

**Figure 1:**
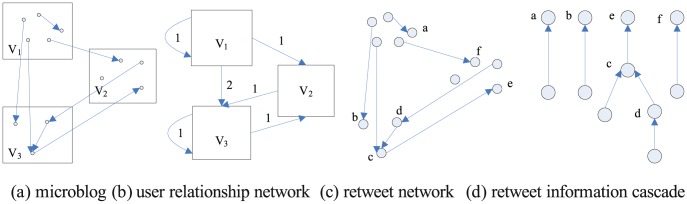
Relationship in microblog.

We build the user relationship network and retweeting network for the microblog. The retweeting network is the topic-based cascade. Fig. 1 presents the two networks.

Fig. 1 (a) describes the microblog relationship, G = (V, E). V is the user and E is the retweet or comment. Fig. 1 (b) is the user relationship network, where the weighted edges represent the number of interactions. The network reflects the users' friends' relationships, but everyone has different interests and individuals will not retweet all of their friends' posts. Thus, the user relationship network cannot reflect true influence and can be used to assist analysis topic-based key people. The retweeting network (Fig. 1(c)) breaks through the friend relationship limitation and solely considers the information cascade (Fig. 1(d)) of retweeting and commenting behavior in a time sequence. An information cascade is also known as a session tree, and the outdegree of its initiator is 0. The other out-degrees link to the initiator or participators to form the information cascade through retweeting, sharing, comments, and so forth. Thus, the outdegrees have the opposite influence of the directed edge. The isolated nodes do not possess retweeted information from others (for instance, the isolated node in Fig. 1(c)). This study considers only the information that can be retweeted at least once.

### Influence index and conformity index

**Figure 2:**
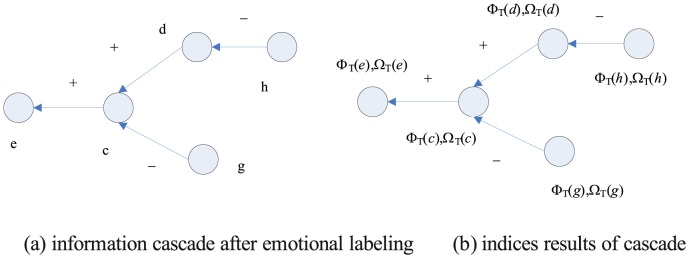
Information cascade after emotional labeling and index calculation.

The influence index measures an individual's capacity to influence others, while the conformity index is the degree to which an individual is affected by others. High-influence individuals' social networks are those whose views and opinions are always accepted, indicating that emotional factors need to be considered in microblog key person extraction. Similar to the behavior of other networks, microblog retweeting mainly includes positive and negative emotions. In Fig. 1 (b), an edge from node a to node b means agree or disagree with b, where the agreement edges are marked as positive (+) or negative (−). Studies have shown that network information dissemination depends on mutual influence among people who are easily affected by others. Thus, the conformity index should be considered along with the influence analysis. Using the emotional inclination analysis method [18], we can find the emotional labeled directed graph G (V, E), where each edge is marked as positive or negative. The emotional labeling of the information is shown in Fig. 2 (a).

*E* ^+^ = {*ce, dc*} indicates positive emotion and *E* ^−^ = {*hd*,*gc*} indicates negative emotion. The G(V,E) is composed of the positive subgraph G^+^(V,E^+^) and the negative subgraph G^−^(V,E^−^). The influence index Φ(*v*) and conformity index Ω(*v*) of node *v* in G are as follows:
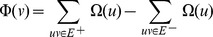


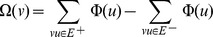



The two indices should be calculated using a recursive loop. All *v*∈*V* in a certain subject T can be initialized by Φ_T_(*v*)  =  Ω_T_(*v*)  = 1. The convergence results are shown in Fig. 2 (b).

### ITEPE Algorithm

The size of the information propagation is determined in the first steps, so the extraction of information initiators and early important participants is important in public opinion analysis. Initiators are those users with the original information, but some users have the habit of directly copying others' blog posts. Thus, these nodes must be deleted using the user relationship network. The term ‘early important participants’ refers to early participants with higher levels of influence. The core idea of ITEPE is shown in Algorithm 1.

Algorithm 1: The ITEPE algorithm

Input: Social Network *G* (*V*, *E*)

Output: Keyperson_T_ for each topic *T*

1) Begin

2)    *C*←**ExtractCascade** (*G*);

3)   IF *C* is context-aware then

4)      *ς*←**ExtractSubgraph**(*C*);

5)    else

6)      *ς* = {*C*}

7)    for each G_T_∈*ς* do

8)   IT_T_←**ExtractInitiator**(*G_T_*)

9)  if G_T_ is not a signed network then

10)       (G^+^_T_(V_T_,E^+^_T_), (G^−^_T_(V_T_,E^−^_T_)) ←**EdgeLabel**(*G_T_*);

11)       (Φ_T_, Ω_T_) ←**IndexCompute**(G^+^_T_(V_T_,E^+^_T_)), (G^−^_T_(V_T_,E^−^_T_));

12)       EP_T_← **EarlyParticipants**(Φ_T_, Ω_T_)

13)       KP_T_←(IT_T_,EP_T_)

14) End

First, the retweeting cascades are extracted from microblog data from defined periods and divided into several cascade sets based on context awareness. Second, true initiators IT_T_ are confirmed for a certain topic T using the user relationship network. The emotional label, influence index and conformity index are then calculated to obtain the early important participants EP_T_. The union set of IT_T_ and EP_T_ is the key person K_T_ for microblog information traceability. The algorithm includes six main parts: ExtractCascade, ExtractSubgraph, ExtractInitiator, EdgeLabel, IndexCompute and EarlyParticipants six main parts.

(1) ExtractCascade is shown in Algorithm 2, where edge E in the social network can be understood as a minimum cascade. The two existing cascades are merged if they intersect. Linear and explosive spreads are two extreme cases, with the actual cascade generally falling somewhere in between.

**Algorithm 2**: The ExtractCascade algorithm.

**Input**: Social network G (V, E), E =  {*e_1_, e_2_, …e_m_*} is the retweet relationship

**Output**: A set of isolated cascades *C* =  {*c* ^1^, *c* ^2^,…, *c^s^*}

**1)** **Begin**

**2)**    initialize each cascade as a single link *C* ← *E*;

**3)** **while** ∃c^p^, c^q^ and c^p^ ∩ c^q^ ≠ Ø **do**

**4)** **forall** *c^i^, c^j^* ∈*C* **and** *c^i^*≠*c^j^* **do**

**5)** **if** *φ^i^* ∩ *φ^j^* **then**

**6)**       add j to i: *c^i^* ← *c^i^, c^j^*;

**7)**       remove j: *C* ← *C*\{*c^j^*};

**8)** **End**

(2) There are many latent semantic mining algorithms that have useful applications for social networking data (e.g., LDA). The microblog contains more obvious topic information, and most microblog posts on the same topic are nearly identical. Thus, the keyword matching method can be used for the topic-based subgraph extraction, the core idea being to cluster the cascade based on the same keywords. For example, topic T has keywords {*k_1_*,*k_2_*,*k_3_*}, and the cascade set G_T_ is composed of the cascades that contain those three keywords.

(3) Based on G_T_, the ExtractInitiator extraction algorithm finds all of the source nodes and the corresponding timestamps to determine an effective initiator. Some users directly copy other users' blog posts, so these nodes must be deleted using the user relationship network. The goal is to analyze the relationships among all of the source nodes and delete the nodes that republish the same topical information to their friends.

(4) *E* =  {*uv*} means *u* retweets *v*, and ∀*E*∈ *c^i^*⊂*G_T_*. E is identified as positive emotions *E* ^+^ with no comments; the emotion word table was used to calculate the emotional inclination of comments [21]. If the comments contain several emotional words, we take the average. Make *E*→*E* ^+^ when the value is greater than 0.5, and *E*→*E* ^−^ otherwise.

(5) If ∀*v*∈*c^i^*⊂*G_T_*,Φ(*v*) = Ω(*v*) = 1, then the influence index Φ(*v*) and conformity index Ω(*v*) are iteratively calculated and normalized.

(6) *T^T^* = Earliest{*T^i^*|*c_i_*⊂*G_T_*} is the timestamp of *G_T_*, ∀*v*∈*c^i^*⊂*G_T_*, and *τ* = Φ(*v*)/Ω(*v*). Thus, the early important participants *v* should satisfy earlier *T^v^* and higher *τ*.

## Experiments

### Dataset

The dataset comes from China's well-known Sina microblog. The Sina microblog opened to the public in October 2009 and today possesses nearly 600 million registered users, nearly 100 million of whom are daily users. Microblog information is characterized by timeliness, and most topics will fade quickly from view. The experimental dataset uses partial data from January 2013 (a total of 85,116,132 posts) for analysis. Because the proposed algorithm analyzes microblog information traceability, the isolated nodes should be deleted. Subjects of blog posts include people's work, the economy, entertainment, education, sports and other fields (e.g., Spring Festival travel was the subject of 4,728,674 posts and haze, the subject of 2,389,428 posts).

### Cascade extraction

**Figure 3:**
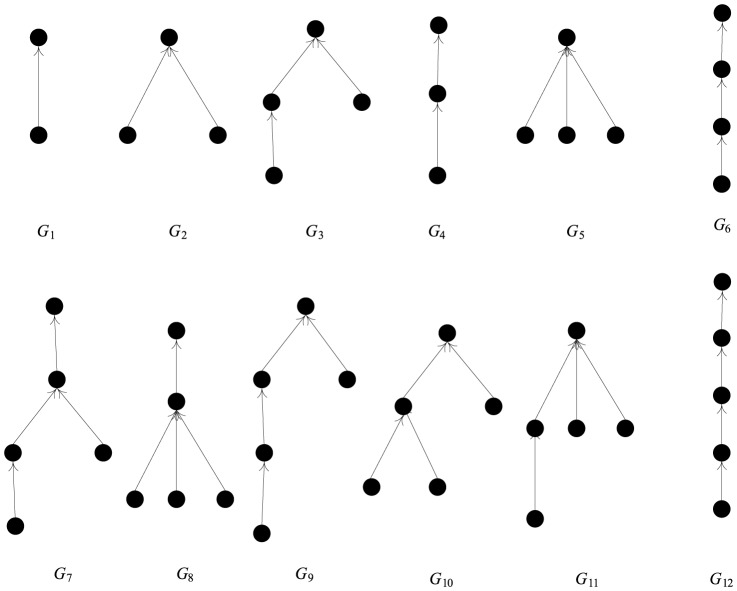
Basic high frequency cascade topology structure.

Although the isolated nodes have been deleted, nearly 76% of the cascade extraction results are less than or equal to 3. Table 2 compares the cascade extraction results of microblogs and blogs [3]. The 204 cascades with different topologies were extracted from the data. The most common cascade is a simple cascade with only one retweet. The 12 highest frequency topology structures are shown is Fig. 3. First, the cascades are arranged in descending order according to the frequency of occurrence (G1 has the highest frequency). Second, the cascade shape is divided into chain and star two categories. The chain with a node in a layer focuses on depth dissemination, and the star with several nodes in a layer focuses on the breadth of spread. In our experiments, the star appears more frequently than the chain in the same cascade scale (e.g., G2 has a higher frequency than G4, and G8 has a higher frequency than G12). Additionally, multiple initiations cannot occur in microblog retweeting, so the cascade topology has only one root node.

**Table 2:** Comparison of cascade extraction data.

Microblog index		Blog index	
Nodes (User ID)	1,824,955	Number of blogs	156,195
Edges (Number of retweets)	96,439	Edges (Number of hyperlinks)	340,124
Number of microblog texts	82,123,008	Number of posts	873,469
Number of cascades	48743	Number of cascades	7,269
Cascades size ≤2	24529	Cascades size ≤2	5674
Cascades size = 3	12472	Cascades size = 3	883
Cascades size ≥3	11742	Cascades size ≥3	712

The microblog cascades that appear in table 2 are also largely simple cascades, but the probability that a cascade forms is higher than a blog, which indicates that the information in a microblog is more fluid. The significant difference in the high frequency simple cascade topology has two main aspects: (1) blogs have more chains and microblogs have more star structures because on a microblog, every piece of information is pushed on to friends, which increases the probability of it being retweeted simultaneously; and 2) blog cascades can contain multiple initiators and blog posts can contain multiple hyperlinks, but retweeting behavior in a microblog setting can only be performed by an initiator.

### Topic-based subgraph extractions

The keyword matching method can complete the topic-based subgraph extraction, the core idea of which is clustering the cascades around the same keywords, i.e., topic T has keywords {*k_1_*,*k_2_*,*k_3_*} and cascade set G_T_ is composed of the cascades that contain those three keywords. For instance, the keywords for January 2013 describe new traffic rules {running yellow lights, new traffic rules, 6-point deduction} and the cascades contain any keywords composed of a new set for information traceability mining. Some particularly widespread events of relatively long durations (e.g., Spring Festival travel and the Spring Festival Gala) will have larger cascade sets. For instance, there are 4,728,674 posts regarding Spring Festival travel, 2,389,428 posts regarding haze and 1,425,510 posts containing the phrase “I am a singer.” This type of information attracts public discussion and comments and the traceability is insignificant. Thus, this paper mainly focuses on emergencies and false information traceability, which are of relatively short duration but have a greater influence on public opinion. Table 3 shows the microblog events set for subsequent traceability research.

**Table 3:** Events and main index.

No.	Microblog events	Version numbers	Retweeting numbers
1	Homeowners series of events cited attention	2812	19991
2	Heilongjiang women's petitions	1427	12797
3	Yuan Longping recommended that the government should treat waste as a criminal act	1347	22218
4	Wenzhou posted two session road closures annunciate cited attention	943	24803
5	Jiangxi Secondary School students punished in playground	742	14510
6	Stewardess purchasing sentenced to 11 years and second trial made controversy	630	11609
7	Hubei Huangshi mobilized preparation the country unannounced inspection 98 days in advance	574	5772
8	Censorship of James Bond film	544	18391
9	Li Yundi was elected as Chongqing CPPCC members	319	12546

The version numbers in the experiments are the number of cascades; a post and its associated retweets form a cascade. Because the same posts are often copied or minimally edited for reposting, the version numbers are generally much larger than the news numbers. True information initiator mining is the first step in traceability studies.

### Initiators

Although they will have the same keywords, posts about an event will focus on various interpretations of it due to different perspectives and the passage of time. *S_i_* is the source node in cascade *C_i_* in topic *T*, and the true information initiators reduce elements in set S. This mainly includes two aspects: (1) cascades of high similarity are classified as one version and only retain the earliest timestamp source node; and (2) the nodes that subsequently publish the same topic information to friends are deleted using the user relationship network. Specific ideas include analyzing the relationship between all of the source nodes and deleting the nodes that subsequently publish the same topic information to friends through horizontal text similarity. Classical text similarity measurement methods include KL relative entropy, TF-IDF, cosine distance, editing distance, and so on. The Sina microblog is similar to Twitter, allowing users to post short messages of up to 140 characters and to obtain followers. The majority of the text of similar short messages does not change, so the simple word repetition rate is used to measure text similarity, as shown in the following equation:
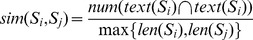
where *len*(*S_i_*) is the number of characters of *S_i_*, *num*(*text*(*S_i_*)∩*text*(*S_j_*)) is the repeated total number of characters of *S_i_* and *S_j_*, and a higher *sim*() value indicates greater similarity, with a value of 1 when a post is copied directly. If *sim*(*S_i_*,*S_j_*) is greater than the threshold 0.71 in the experiment, cascades *C_i_* and *C_j_* can be about a similar message. Table 4 shows the news version number of 9 popular microblog issues, which is far lower than the number of cascades. To mine the true information initiator, the source nodes with the earliest timestamps of every new version are examined. For instance, there are 734 versions of the No. 1 issue, which means that there are 734 possible initiators. Thus, the user relationship should be analyzed further. If a user's message is similar to that of his or her friend's earlier messages, the user can be identified as a false initiator. If *sim*(*S_i_*,*S_j_*) is greater than the threshold 0.47 in the experiment and the timestamp is later than that of a friend's, then the source node will be identified as a copier. The initiators of the different issues are all within 80 in Table 4 compared to the tens of thousands of microblog information disseminators. Thus, this magnitude benefits traceability and the control of public opinion.

**Table 4:** Cascade set of issues.

No.	1	2	3	4	5	6	7	8	9
Cascade numbers	2812	1427	1347	943	742	630	574	544	319
Version numbers	734	254	240	334	659	226	239	289	221
Initiators	63	53	67	71	24	36	43	29	58

A special phenomenon common to microblogs cannot be ignored: if a highly influential node retweets a message that is then widely retweeted, then the participants are more important than the initiators. Therefore, only mining the initiators in the information traceability is insufficient.

### Early important participants

**Figure 4:**
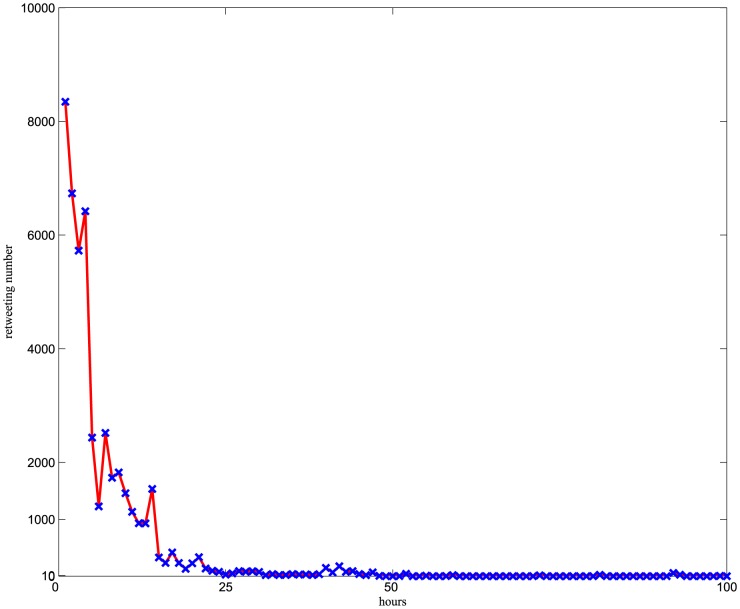
Retweets in a cascade over time.

The time factor is very important in information traceability studies, whereas the average new edges of every node changes little over time and the cascade edge generation decreases exponentially [9]. Experiments show that the user relationship network is in line with the former, whereas users participating in a cascade follow the latter. Fig. 4 shows that the number of retweets together with the corresponding time of the largest cascade in the number 1 issue.

Eighty-four percent of retweets are posted within 10 hours, which also highlights the rapidity of information transmission on microblogs. To identify the source accurately, the influence index and conformity index of every node will be iteratively calculated according to the text sentiment analysis and information cascade, following which the early important participants will be extracted. The early important participants have the following characteristics: (1) earlier participant time; and (2) high influence index and low conformity index.

The emotional terms and phrases were counted in the comment data [18], including both supportive words and opposing words, and the 30 items with the greatest frequency were selected. The negative terms were valued between 0.5 and 1, with larger values indicating greater opposition; likewise, the positive terms were valued between 0 and 0.5, with smaller values indicating greater support. E was identified as positive emotions *E* ^+^ with no comments, and the emotion word table was used to calculate the emotional inclination of comments. If the comments contained several emotional words, we took the average. *E*→*E* ^+^ if the value is greater than 0.5; Otherwise *E*→*E* ^−^.

**Figure 5:**
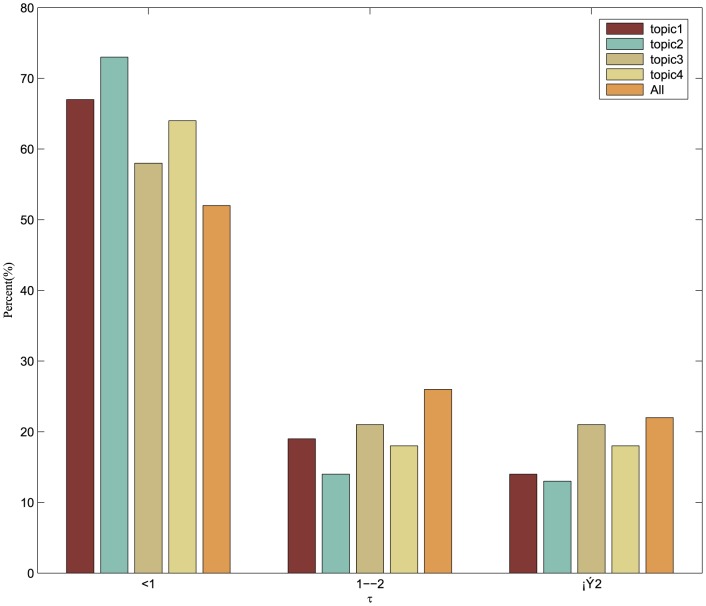
Comprehensive influence distribution in various topics.

For any *v*∈*c^i^*⊂*G_T_*, let Φ(*v*)  =  Ω(*v*)  = 1. The influence index Φ(*v*) and conformity index Ω(*v*) are then iteratively calculated and normalized. The *τ* =  Φ(*v*)/Ω(*v*) is defined to measure the user comprehensive influence, and the users' *τ* distribution across different topics is shown in Fig. 5.

The comprehensive influence indices *τ* for all 9 issues were analyzed. Fig. 4 presents four issues and their averages. *τ*≥2 means that the influence index Φ(*v*) is significantly greater than the conformity index. The partial users, who make up less than 20%, can influence other users and cannot easily be affected. This indicates that there a few people who can affect the information dissemination model of the majority on microblogs.

**Figure 6:**
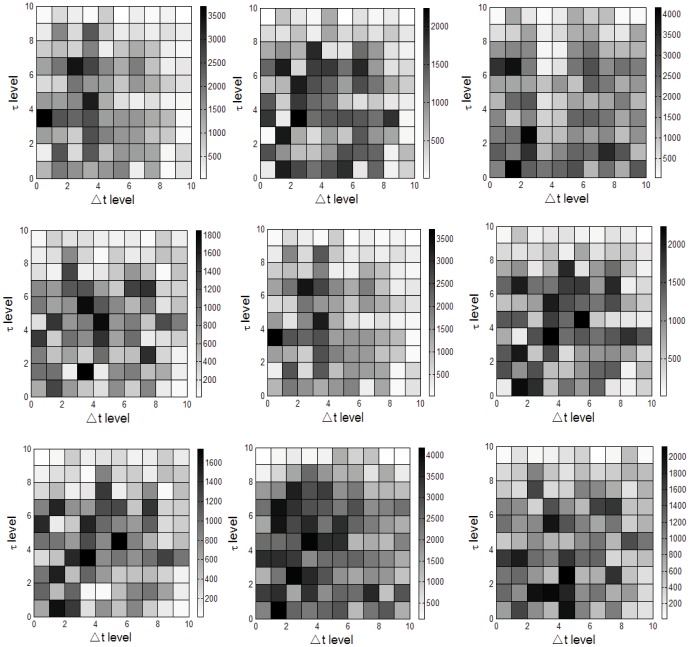
User influence and participation time statistics.

The time factor is important in information traceability studies. Early important participant *v* should satisfy an earlier timestamp *T^v^* and higher comprehensive influence index*τ*. The *T^T^* =  Earliest {*T^i^*|*c_i_*⊂*G_T_*} is defined as the timestamp of *G_T_*. The T^*^ =  Latest {*T^i^*|*c_i_*⊂*G_T_*} is defined as the latest timestamp of *G_T_*. For any∀*v*∈ *c^i^*⊂*G_T_*, *T^v^* is the timestamp of *v* participating in cascade *c^i^*. Thus, *t* = *T^v^*–*T^T^*. To find the early important participants with a small △*t* and large*τ*, the time and comprehensive influence of the users involved in different issues are analyzed in Fig. 6.

Because different issues have different scales over time, the quantitative classification method is adopted for comparison. Δ*t* level divides the total during time T^*^–*T^T^* into 10 sections, and the comprehensive influence level divides the difference in influence in the issue into 10 sections where for any *v*∈ *c^i^*⊂*G_T_*, *τ_max_* = *max*(*τ_v_*),*τ_min_* = *min*(*τ_v_*). Thus, the coordinates of *v* are (10(*T^v^*–*T^T^*)/(T^*^–*T^T^*), 10(*τ_v_*–*τ_min_*)/(*τ_max_*–*τ_min_*)). Additionally, a deeper color indicates that more users have different magnitudes through different issues.

It can be seen that most of the events have more active first halves and less active second halves. Some events have a second peak in the number of participants, which may be due to the propagation of a new message that reignites the issue. The users with high comprehensive influence levels are a minority, particularly in the 9^th^ and 10^th^ levels. The nodes in the upper left corner of the first square are the early important participants with the highest *τ* level and lowest Δ*t* level, and the maximum value in all 9 topics is 101.

### The assessment and validation of traceability results

Early important participants may include some initiators, so the results are the union of both. The results of all of the events in Table 3 are shown in Table 5. Thus, the information traceability results are basically locked within 131 IDs that reached the mining results. To evaluate the accuracy of the results, the following aspects must be considered: (a) whether the result set contains the earliest initiator; (b) whether a friend relationship exists between the nodes; (c) manual analysis of whether tweeted text is related to the event; and (d) whether the tweet and follower numbers are collected to analyze if they are the active nodes. √ means that the traceability results satisfy the index, and × means that the traceability results do not satisfy the index.

**Table 5:** Results of ITEPE.

No.	1	2	3	4	5	6	7	8	9
Initiator	63	53	67	71	24	36	43	29	58
Early important participants	86	74	101	81	61	50	39	57	37
Source nodes	120	113	104	131	74	71	69	78	85
(a)	√	√	√	√	√	√	√	√	√
(b)	×	×	×	×	√	×	×	×	√
(c)	√	√	√	√	√	√	√	√	√
(d)	√	√	√	×	√	√	×	√	√

All of the events that reached Index (a) show that the traceability nodes contain the initiator, which also illustrates that the true information initiator is not to be missed. Finding the earliest initiator is required by the conventional sense source tracing method. From the above analysis, it can be seen that only finding one node in the traceability process is insufficient. For Index (b), if there are friends in the same cascade, then the nodes that subsequently publish the same information to their friends will be deleted, so the source nodes in the majority of the events are isolated nodes without friendship ties. However, friendships appear in the 5^th^ and 9^th^ issues because more than half of the subsequent messages were altered for posting. Thus, these nodes are retained to carry new information. All of the events satisfy Index (c) that tweeted text is related to an event, which is determined by the algorithm characteristic because the topic and cascade locked in by the keywords lessen the possibility of topic drift. Index (d) measures the activity of the nodes and the active nodes with more followers and larger tweet numbers. Only the 4^th^ and 7^th^ issues have minority inactive nodes. The inactive nodes are easily eliminated in the cascade extraction process because those messages have a smaller chance of being seen by others. Sometimes inactive nodes are independent-minded or well informed, such as a military enthusiast posting a new message to a microblog even though he does not usually use this kind of platform. In summary, the traceability results contain the earliest initiator, and most nodes are active. The algorithm controls topic drift and ensures accuracy.

## Conclusions

Microblog source tracing must quickly lock in the information source, which is critical to public opinion analysis and early warning. To confirm these important nodes, ITEPE is proposed. First, the cascade (session tree) is built according to retweets in microblogs, and the cascade set (session forest) is clustered by topic relevance. Second, real initiators are identified through the user relationship network and information cascade network. The influence index and conformity index of every node is then iteratively calculated according to the text sentiment analysis and information cascade, and the early important participants are extracted. Finally, the real initiators and early participants are identified and evaluated through an experiment. Analysis of data from the Sina microblog from January 2013 yielded 9 popular events. The source tracing process combines news features, microblog information propagation characteristics, textual emotional features and user characteristics to analyze and evaluate, and the result set has high accuracy.
